# Recent Advances in Nanocarrier-Assisted Therapeutics Delivery Systems

**DOI:** 10.3390/pharmaceutics12090837

**Published:** 2020-09-01

**Authors:** Shi Su, Peter M. Kang

**Affiliations:** Cardiovascular Institute, Beth Israel Deaconess Medical Center and Harvard Medical School, 3 Blackfan Circle, CLS 910, Boston, MA 02215, USA; ssu@bidmc.harvard.edu

**Keywords:** nanomedicine, nanocarriers, drug delivery

## Abstract

Nanotechnologies have attracted increasing attention in their application in medicine, especially in the development of new drug delivery systems. With the help of nano-sized carriers, drugs can reach specific diseased areas, prolonging therapeutic efficacy while decreasing undesired side-effects. In addition, recent nanotechnological advances, such as surface stabilization and stimuli-responsive functionalization have also significantly improved the targeting capacity and therapeutic efficacy of the nanocarrier assisted drug delivery system. In this review, we evaluate recent advances in the development of different nanocarriers and their applications in therapeutics delivery.

## 1. Introduction

Nanotechnology has emerged to be an area of active investigation, especially in its applications in medicine [1]. The nanoscale manipulation allows optimal targeting and delivery as well as the controllable release of drugs or imaging agents [2]. Among all the applications of nanotechnology in medicine, nanocarrier assisted drug delivery system has attracted significant research interest due to its great translational value. The small size of the nanocarriers can help drugs overcome certain biological barriers to reach diseased areas [3,4]. Taking advantage of different nano-sized materials and various structures, nanocarriers can help poorly soluble drugs become more bioavailable and protect easily degraded therapeutics from degradation [5,6]. In addition, the modifiable surfaces of nanocarriers also expand their usability in different biomedical applications, especially in targeted therapy [7]. Indeed, their modification can not only stabilize but also functionalize them to be responsive to different stimuli, improving the therapeutic efficacy [7]. Herein we review recent advances in the development and applications of various nanocarriers, discussing their advantages and disadvantages in terms of their different compositions as well as different functionalization techniques. As the nanocarrier assisted drug delivery system is a broad field under active investigation, in order to provide a more in-depth review, after an overview of different types of nanocarriers, we will focus on stimuli-response nanocarriers.

## 2. Methods

This review was written in compliance with the preferred reporting items for systematic reviews and meta-analyses (PRISMA) protocol [8]. We used PubMed as our database, and searched original research articles and relevant reviews by entering keywords such as “nanomedicine” and “drug delivery.” Search results were critically analyzed and categorized by the nature of the drug delivery system.

## 3. Types of Nanocarriers

**Figure 1:**
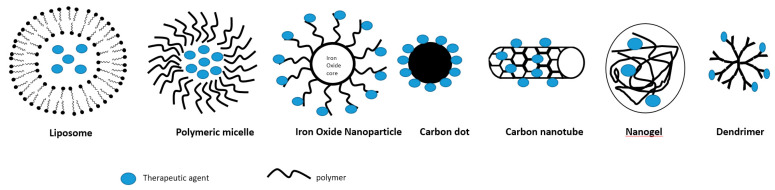
Schematic illustration of some nanocarriers.

Nanocarrier assisted drug delivery systems have gained increasing recognition in recent years for biomedical applications. As different clinical needs require different types of drug delivery systems, various designs of nanocarriers are being developed in order to meet specific requirements. There are a variety of nanocarriers for the drug delivery categorized by different compositions and structures, including carbon nanotubes, carbon dots, polymeric micelles, liposomes, iron oxide nanoparticles, nanogels, and dendrimers [7,9] (Figure 1).

### 3.1. Liposomes

Liposomes are phospholipid vesicles consisting of lipid bilayers enclosing discrete aqueous spaces [10,11]. Several features of liposomes make them good candidates as drug delivery systems. These self-assembled nanocarriers are biocompatible, easily modifiable, and capable of carrying large drug payloads [10]. Liposomes are able to entrap both lipophilic and hydrophilic compounds (drugs and/or imaging agents), in the lipid membrane and the aqueous core, respectively [12]. They are also generally considered to have a good safety profile [10]. Conventional liposomal nanocarriers are simple self-assembled lipid bilayers carrying therapeutics in their aqueous cores. The lipid bilayers can be further stabilized by adding polyethylene glycol (PEG) to the surface, a modification called PEGylation [13]. Liposomes can also be functionalized by modifying the surface with specific ligands [10]. Furthermore, they can be equipped with imaging agents on the surface together with the targeting ligand to improve targeting efficiency while possessing both therapeutic and diagnostic (theranostic) properties [10]. The major biological challenge of liposomal carriers is their fast clearance by the reticuloendothelial system (RES) [10]. Even though surface modification of liposomes by PEGylation can significantly minimize their uptake by the RES, the clearance cannot be completely avoided [10]. Constant modifications have also been made in order to improve the targeting efficiency and therapeutic efficacy of liposomes while decreasing their toxicity [10]. In order to overcome the challenge of being quickly cleared by the RES, Tang et al. reported a new modification to the surface of the liposome with a “do not-eat-me” strategy [14]. Inspired by the findings that the expression of CD47 is upregulated on the surface of certain cancer cells to avoid phagocytosis, this strategy was achieved by adding a CD47-derived, enzyme-resistant peptide to the surface of the liposome in order to block the RES so that the circulation time of the liposome can be prolonged [14,15].

Liposomal carriers have been widely used as nanocarriers for drug delivery as they have shown significant improvement in therapeutic efficacy by stabilizing the payload and assisting targeted tissue uptake [10,11,16]. The Food and Drug Administrations’ (FDA) first approval of Doxil, a liposomal drug carrying an anti-cancer agent- doxorubicin hydrochloride, has paved the way for the clinical translation of nanocarriers [9]. In subsequent years, liposomes continue to be the dominant nanocarrier among all the nanocarrier-assisted drug submissions to the FDA, suggesting the safety and effectiveness of this type of nanocarrier [16]. Thus far, the majority of liposomal nanocarriers are intended for intravenous use [16]. While being the most extensively used nanocarriers in cancer therapy, wider applications of liposomal nanocarriers are still challenged by their capacity to reach the desired delivery efficiency and their inevitable off-target accumulations [17].

In addition to drug delivery, there is also active research exploring the potential of liposomal nanocarriers for nucleic acids delivery [18,19,20]. One major hurdle for targeted therapy using liposomal nanocarriers delivering nucleic acids is that systemic administration of these nanocarriers leads predominantly to hepatic uptake [21]. In order to improve the tissue specificity of the liposomal carriers, tissue specific antibodies can be incorporated. For example, in order to achieve the delivery of transgenes to the pulmonary vasculature, Parhiz et al. recently conjugated the mRNA-carrying liposomal nanocarriers with antibodies specific to the vascular cell adhesion molecule, PECAM-1 [21]. This novel approach profoundly reduced the hepatic uptake and helped guiding the nanocarriers to the desired organs [21]. Using a similar approach, Marcos-Contreras et al. developed a liposomal nanocarrier conjugated with antibodies that are specific to the endothelium while carrying the mRNA of thrombomodulin, a natural endothelial inhibitor of thrombosis, inflammation, and vascular leakage [22]. These liposomal mRNA nanocarriers were able to target the inflamed cerebral vasculature and alleviate TNF-induced acute brain inflammation [22].

### 3.2. Biodegradable Polymeric Micelles

Because of the toxic effects elicited by the metal nanoparticles, synthetic nanoparticles composed of biodegradable nanomaterials are generally more favorable [23]. A variety of biodegradable polymers are available for the development of nanocarriers, including polylactic acid (PLA), poly(lactic-co-glycolic acid) (PLGA), polycaprolactone (PCL), as well as their modifications and/ or combinations [24,25]. Such biodegradable polymers can be used as blocks to form micelles, a type of self-assembled nanovesicle with a hydrophilic outer layer and a hydrophobic core that can encapsulate drugs with low solubility [25,26,27].

Different approaches have been investigated to improve the drug loading capacity of biocompatible polymeric nanocarriers, including the incorporation of albumin. Such modification led to the development and eventually FDA approval of an albumin-based polymeric nanocarrier, Abraxane, which carries an anti-cancer drug, Paclitaxel, for the treatment of breast cancer [28]. Other incorporations include milk protein casein, elastin-like peptide, and DNA origamis as components of the drug delivery nanocarriers [28]. Surface functionalization of the micelles can also help in protecting payloads such as protein drugs from being degraded in the gastrointestinal system, making it possible for oral delivery of protein drugs such as insulin [6,29]. Recently, Han et al. developed a modified micelle platform featuring a virus-mimetic zwitterionic surface that enables drug penetration through the mucus and efficient transporter-mediated epithelial absorption without the need for tight junction opening [30]. This platform could reach over 40% bioavailability of orally delivered insulin, paving the way for a potential safe and painless delivery method for insulin [30].

Efforts have also been made to improve the drug-releasing profile of polymeric micelles. Recently, Schudel et al. developed a programmable multistage drug delivery system targeting at the lymph nodes [31]. They synthesized polymeric micelles that can be easily taken up by lymph nodes and added thiol-reactive oxanorbornadiene (OND) linkers to these nanoparticles [31]. Depending on different OND substituents, the half-life of the nanocarrier can be programed ranging from hours to days [31]. Such multi-staged intra-lymphatic drug release can help in augmenting the immunotherapeutic effects compared to free drug delivery [31].

Some of the efforts to prolong the circulation period of micelles resulted in the development of one subset of micelles called filomicelles. Filomicelles are flexible and fragmentable filamentous micelles made of degradable block copolymer amphiphiles [32]. The inspiration for filomicelles came from filoviruses and tubular proplatelets that break up into smaller platelets in blood flow [32]. Shuvaev et al. developed targeted filomicelles conjugated with antibodies that recognize distinct endothelial surface molecules [33]. These targeted filomicelles were able to not only retain structural integrity and dynamic flexibility but also adhere to endothelium with high specificity both in vitro and in vivo [33]. These results provided the basis for a new drug delivery approach employing antibody-targeted filomicelles that circulate for a prolonged period of time while being capable of binding to endothelial cells in vascular beds expressing certain markers [33].

As demonstrated in recent research advances, therapeutics conjugated with biodegradable polymers offer several advantages, including improved drug solubilization that prolongs therapeutic half-life, reduced immunogenicity to enhance safety, and better-controlled drug release [34]. However, several challenges remain to be overcome. For example, when polymers are conjugated with proteins, they can reduce the bioactivity of protein therapeutics [34]. In the field of polymers conjugated with small molecules, very limited drugs achieved translational success [34]. The huge gap between the preclinical studies and human results remain to be filled [34].

### 3.3. Carbon Nanotubes

Carbon nanotubes (CNTs) are carbon allotropes possessing a tube-like hollow cylindrical structure [35]. Based on the layer of the tubes, CNTs can be classified as either single-walled (SWNTs) (1 nm diameter) or multi-walled (MWNTs) (5 to 20 nm outer and 2 to 6 nm inner diameter) [35]. Raw CNTs are hydrophobic [36]. Therefore, in order for the CNTs to be appropriate for biomedical use, they first need to be functionalized to be water-soluble and biocompatible [36]. Certain functionalization, such as PEGylation, can significantly improve the drug loading capacity of the CNT [37]. Aromatic molecular drugs such as Doxorubicin can bind to the CNT surface through supramolecular π–π stacking [36,37]. Functionalized CNTs can act as carriers for various therapeutics (from small-molecules to peptides, to nucleic acids) and are able to deliver the active agents to various organs depending on the functionalization of the nanotubes, which may also be responsive to different stimuli [35,36,38].

Besides their wide application in cancer therapy, the potential application of CNTs in the treatment of other diseases is being explored as well. Leeper’s group has recently used PEG-functionalized SWNTs loaded with a fluorescent probe and a small-molecule inhibitor of the anti-phagocytic CD47-SIRPα signaling axis in order to prevent atherosclerosis [39]. Being capable of easily penetrating the cells, CNTs hold certain potential of crossing the blood-brain barrier (BBB) to treat neurological diseases [40]. Porter’s group found that functionalized anionic MWNTs have the highest transportation rate across the human BBB [40].

In terms of the safety profile of the CNTs, studies suggested that endocytosis of the CNTs induces oxidative stress to the cells, revealing a close connection between CNTs and inflammation, fibrosis, and cancer, impeding the translational value of this nanocarrier [41,42]. In addition, the toxicity of CNTs is not only related to their shape but also to their surface charge [40,43,44,45,46].

### 3.4. Carbon Dots

Carbon dots (CDs) were serendipitously discovered by Xu et al. during the purification of single-walled CNTs [47]. Subsequently, Sun et al. prepared carbon nanoparticles with luminescence emission across the visible range and near-infrared region [48]. It was then that the term “carbon dots” was coined and used to identify fluorescent carbon nanoparticles. CDs are therefore the newest addition to the carbon family. Surface functionalization of CDs allows them to be used for different biomedical purposes, including bio-imaging and drug delivery [49]. Drugs can be conjugated to the CDs through non-covalent bonding utilizing the carboxyl group on CDs or through electrostatic interactions via the functional groups [50,51]. CDs enter cells through endocytosis and passive diffusion, and the conjugated drugs are subsequently passively released inside the cells [52].

The size of CDs is less than 10 nm [49,53]. Due to their particularly small size, CDs provide hopes to overcome the challenge of delivering drugs across the BBB for the treatment of neurological diseases. Leblanc’s group developed carbon dots conjugated with targeting ligand and therapeutic drugs in order to treat glioblastoma brain tumors [53]. They subsequently developed carbon nitride dots for their potential use in pediatric glioblastoma treatments [54]. They tested the drug in the zebrafish model to demonstrate the carbon nitride dots were capable of BBB penetration [54]. However, these carbon dots still need further validation for their safety profile [55,56].

### 3.5. Iron Oxide Nanoparticles

Due to their biocompatibility, relatively low toxicity, and their ability to randomly flip direction of magnetization under the influence of temperature, a feature called “supramagnetism,” iron oxide nanoparticles have widely been used as contrast agents and drug carriers [57]. Iron oxide nanoparticles can be induced into magnetic resonance by self-heating or external magnetic field [57]. Bare iron oxide nanoparticles tend to agglomerate and result in the uptake and clearance by the RES [57]. This challenge can be overcome by coating the nanoparticles with stabilizing nanomaterials, such as PEG, gelatin, or chitosan [57]. Surface functionalization can also help drug release in response to various stimuli [58]. Recently, Zanganeh et al. reported a previously unknown benefit of iron oxide nanoparticles [59]. They found that ferumoxytol, an existing FDA-approved iron oxide nanoparticle used to treat iron deficiency anemia, can inhibit tumor growth by inducing pro-inflammatory macrophage polarization in tumor tissues [59]. The prophylactic use of iron oxide nanoparticles in vivo was also shown to be capable of preventing the development of hepatic metastasis [59]. Their findings suggested an “off-label” use of the ferumoxytol for cancer patients [57]. However, the concern of using iron oxide remains as it has been shown to contribute to the generation of free radicals in the body [57,60,61,62].

### 3.6. Nanogels

Nanogels are nanoscale hydrogels composed of cross-linked swellable polymeric particles [63]. They are mostly hydrophilic, highly biocompatible, and possess high water content [63,64]. Nanogels are considered promising drug delivery systems due to their many advantages, including high drug encapsulation capacity, uniformity, tunable size, ease of preparation, minimal toxicity, stability in the presence of serum, and stimuli responsiveness [65]. Different designs have been incorporated for the development of targeted drug delivering nanogels [66]. Nanogels can release drugs either by passive diffusional release or when the structures with functionalized components are biologically or chemically degraded [66]. However, obstacles also exist for the clinical translation of nanogels. For example, when deformity occurs, their previously advantageous properties such as their swellability, drug loading capacity, and affinity target adhesion can be largely affected [67]. Constant efforts are also being made to optimize their biodistribution, to avoid their fast clearance, to minimize the toxicity caused by the surface charge, and to gain better control of targeted drug release and the degradation of nanogels [64,65]. Working towards these goals, recently, Myerson et al. developed a cross-linker modulated nanogel tunable in shape and equipped with antibodies specific to endothelial markers for improved targeted drug delivery [67].

### 3.7. Dendrimers

Dendrimers are three-dimensional, branched, polymeric architectures in nanoscale [68,69]. The drugs can either be physically entrapped in a dendrimer using non-covalent interactions or covalently coupled on dendrimers [70]. The entrapment and release of the drug can be controlled by modifying the surface of dendrimers [70]. Functionalization of the surface of the dendrimers by attaching targeting ligands can also improve their targeting efficiencies [70]. The dendrimers hold great potential for biomedical applications because of their capacity to entrap high molecular weight hydrophilic or hydrophobic entities, also due to their high surface to volume ratio that enables them to serve as potential gene therapy carriers [69]. They also enhance the solubility, stability, and oral bioavailability of many drugs [70]. Recently, Pan et al. developed a polyamidoamine dendrimers-based nanomedicine that incorporates a small interfering RNA (siRNA) that can downregulate a multidrug resistance (MDR)-related protein onto the dendrimer in order to overcome multidrug resistance which is frequently encountered in cancer treatments [71]. Such advances in technology bring hope to the future of cancer treatment. However, currently, the translational applications of dendrimers have been limited because cationic dendrimers tend to interact with cell membranes and cause cell lysis [69]. Efforts such as carboxylation, PEGylation, or acetylation of terminal amine groups on dendritic polymers have been made in order to decrease the toxicity of dendrimers [72]. Some other limitations of dendrimers include fast clearance from the body and relatively short circulation time, all of which are getting improved with recent technical advancements [72].

### 3.8. Hybrid Nanocarriers

As technology advances, hybrid nanocarriers are also being developed. For example, carbon-based hybrid nanogels provide a platform for drug delivery [73]. The fluorescent carbon dots can not only serve as crosslinkers to stabilize the nanogel, but also provide stable fluorescent signals for real-time imaging [74]. Other hybrid nanogels, such as with liposomes or polymers, are also being developed to increase the biocompatibility of the nanocarriers [75]. Different combinations of nanomaterials are being explored for the best drug-delivery efficacy. Another common example is the combination of polymers and liposomes. Based on this strategy, Cao et al. recently developed a hybrid nanocarrier that encapsulates hydrophobic HIV drugs into PLGA polymeric cores, which is then inserted into lipid bilayers [76]. This hybrid nanocarrier was also conjugated with different antibodies to target CD4^+^ T cells [76]. This strategy significantly improved the latency and decreased the toxicity of some HIV drugs [76].

## 4. Strategies to Enhance Drug Delivery Efficiency

**Figure 2:**
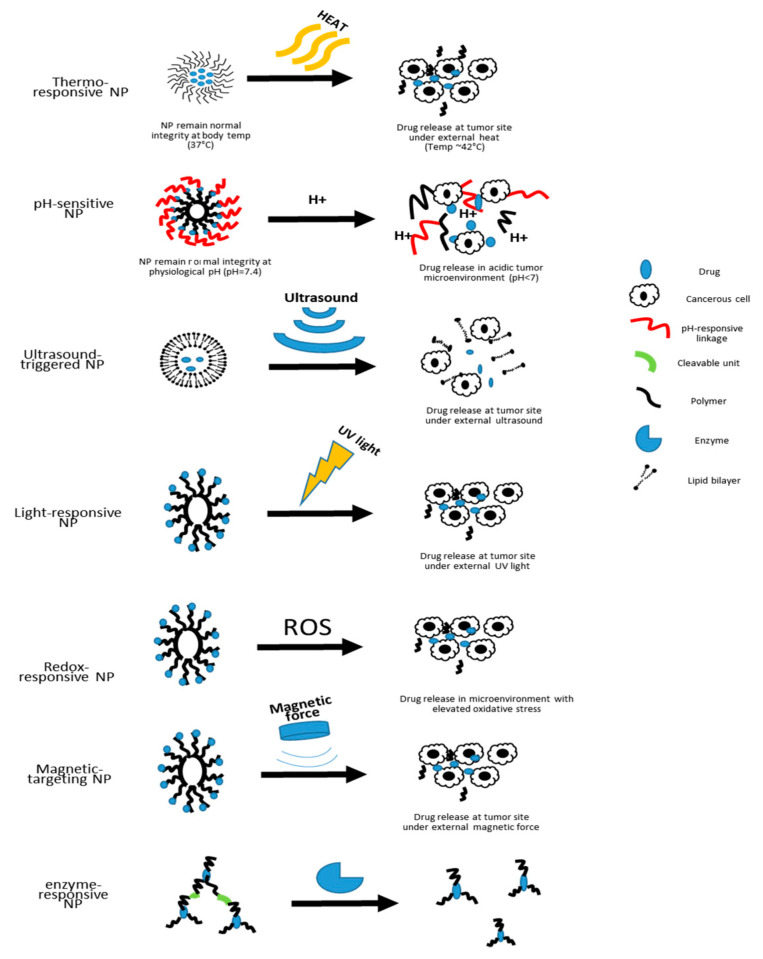
Schematic illustration of the drug-releasing mechanisms of different types of nanocarriers.

As summarized in Table 1, while each nanocarrier possesses unique advantages, they also face certain challenges that limit their optimal performance in the drug delivery system. Without specific targeting functionalization, most nanocarriers depend on their small size to reach the disease site by passive accumulation and retention [77]. Although this system is already superior compared to other drug delivery systems, much optimization still needs to be done in order to achieve optimal therapeutic outcomes, namely targeted drug release at controllable rates [78]. In nanomedicine, one common route for the carried-on drugs to reach the diseased area is to passively diffuse out of the nanocarriers that have accumulated in the diseased tissue [79]. As for adding targeting capacity to the nanocarriers, especially in cancer therapy, a common approach is surface addition of ligands that are specific to receptors overexpressed in certain cancer cells [55]. In an effort to develop smarter nanocarriers that can further improve the targeting efficiency and on-demand drug release, various stimuli-responsive nanocarriers are currently under development [9]. In the following sections, we will discuss the features and applications of some of the stimuli-responsive nanocarriers (Figure 2).

Currently, the stimuli-responsive nanocarriers can be activated by either exogenous stimuli, such as variations in temperature, magnetic field, ultrasound intensity, light or electric pulses, or endogenous stimuli, such as changes in pH, enzyme concentration or redox gradients [9]. The specific design of nanoscale stimuli-responsive systems enables the controlled drug biodistribution in response to specific exogenous or endogenous stimuli [80]. Through the stimuli responsiveness, on-demand drug release could be achievable [80,81,82].

### 4.1. Thermo-Responsive

Thermo-responsive nanocarriers are among the most investigated nanocarriers in cancer therapy for solid tumors [80]. The working concept is that thermo-responsive nanocarriers are able to retain their payload at 37 °C, the physiological body temperature, but rapidly release their payload at the heated tumor area (~40 to 42 °C) [80,83]. As nanotechnology advances, polymeric nanocarriers that exhibit lower critical solution temperature (LCST) and upper critical solution temperature (UCST) can have better control of drug release [84]. This strategy is helpful in the treatment of solid tumors as drugs can be loaded at 37 °C, and depending on the LCST or UCST, drugs can then be released by either cooling or heating the tumor after injection [84]. Based on the existing thermo-responsive technology, an additional pulsatile drug delivery system has been introduced by embedding thermo-responsive polymers to liquid crystals to achieve programmed drug release in concordance with the circadian rhythm [85].

### 4.2. pH-Responsive

As pH values vary in different biological compartments, pH-responsive nanocarriers will allow better control of site-specific drug release [86]. There are two main strategies that exist for the development of pH-sensitive nanocarriers. One is using polymers functionalized with ionizable groups that can undergo a conformational change upon encountering environmental pH change, and the other is the incorporation of acid-sensitive bonds that break in an acidic environment for drug release [80,87]. Due to the acidic microenvironment of tumor sites, pH-responsive nanocarriers rise to be a useful strategy for targeted cancer therapy [88]. The intracellular pH values also differ from the ones in the extracellular matrix [89].

A wide range of nanocarriers can be equipped with pH-responsivity [90]. For example, vaccines for hepatitis B virus are under development using pH-responsive liposomes to achieve cytosolic drug release [91]. Polymeric micelles that are functionalized to be pH-responsive are under active investigation for their application in chemotherapy [92,93]. To overcome certain disadvantages of some pH-sensitive polymers, such as uncontrolled drug-loading or drug-releasing rate as well as undesired toxicity, non-polymer pH-sensitive carbon dots were also developed for cancer therapy [94]. In addition, recently surface modified pH-responsive SWCTs have also been developed to co-deliver anti-cancer drugs and genes [95].

### 4.3. Ultrasound-Triggered

The ultrasound-triggered drug-releasing approach is an appealing on-demand drug-releasing strategy because of their non-invasiveness as well as the controllable frequency and duration in order to regulate the depth of tissue penetration [80]. Ultrasound generated mechanical forces can transform nanodroplets (such as liposomes) to nano-bubbles [80]. These ultrasound-genic nanobubbles increase the efficiency of delivering payloads to neighboring cells [80]. Prabhakar et al. have recently developed a nanobubble liposome complex that can be ultrasound triggered to deliver both imaging agents and anti-cancer drugs, suggesting a promising future for the theranostic application of ultrasound-triggered nanocarriers [96]. However, despite the fact that ultrasound is non-invasive, the frequency applied in order to trigger drug release differs from conventional settings for clinical imaging purposes. Such differences raise the concern of potential mechanical induced cell damage [97]. Another limitation is that the ultrasound beam may be attenuated by the hard tissues and certain tissue associated movements [98].

### 4.4. Light-Responsive

Another non-invasive and controllable approach for drug release is to incorporate light-responsive materials in the nanocarriers. Under certain wavelengths of light, these nanocarriers can either disassemble for drug release or shrink in size for deeper tissue penetration [80]. Wang et al. developed a near-infrared (NIR) light-responsive polymeric nanocarrier by incorporating selenium that can rapidly dissociate within minutes post NIR light exposure due to reactive oxygen species (ROS)-mediated selenium oxidation [99]. Such irreversible dissociation of nanocarriers promotes continuous drug release [99]. With the help of light-responsive nanomaterials, a high degree of spatiotemporal precision can be achieved, but the safety of light-responsive nanocarriers are still not well defined [100,101]. Specifically, the irreversible change of these nanocarriers raises concerns for the safety of byproducts [100].

### 4.5. Redox-Responsive

Given that oxidative stress has been found to be elevated in the pathogenesis of many diseases, another type of stimuli-responsive nanocarriers that have attracted significant interest are the redox-responsive nanocarriers [102,103,104]. The tumor microenvironment has certain features that are different from healthy tissues. For example, there is a significant elevation in the concentrations of glutathione and ROS in tumor microenvironments than in normal tissues [105]. With the help of redox-responsive polymers, these nanocarriers can significantly increase the concentration of drugs released in the diseased area [105,106].

Similarly, in diabetes, hyperglycemia induces cellular hypoxia through mitochondrial ROS production [106,107]. In order to benefit diabetic patients, Gu’s group has been advancing the painless microneedle patch using hypoxia-responsive nanoparticles that release insulin in a hypoxemic microenvironment. They first developed microneedle-array patches loaded with hypoxia-sensitive vesicles to provide fast glucose-responsive insulin delivery [108]. Subsequently, they improved their nanoparticles using H_2_O_2_-responsive polymeric vesicles integrated with transcutaneous patches for glucose-mediated insulin delivery [109]. Combining both technologies, they developed a new nanovesicle that is responsive to both hypoxic environment and H_2_O_2_ in order to enhance insulin delivery [110].

As ROS and redox signaling also play important roles in ischemia/reperfusion (I/R) injury, redox-responsive nanocarriers delivering anti-oxidant to the injured site could also potentially ameliorate I/R injury in the tissues [111,112,113]. For this purpose, Kang and colleagues have developed ROS-responsive nanocarriers for their application in various I/R injuries [114,115,116,117,118]. Recently, Elkassih et al. also developed degradable redox-responsive disulfide cross-linked nanogel drug carriers to decrease cytotoxicity and to increase drug uptake in areas with increased oxidative stress [119].

### 4.6. Magnetic Targeting

Nanocarriers can also be modified to be responsive to magnetic force. In this approach, the therapeutic agents are attached to or encapsulated in magnetic nanocarriers, which are often made of functionalized polymers [120]. Among all the candidate nanomaterials, the biocompatible superparamagnetic iron oxide nanoparticles (SPIONs) with modifications are the most widely used as part of the magnetic nanocarriers [121]. These nanocarriers are then injected into the bloodstream near the target site [120]. When the magnetic fields are applied over the target site, the magnetic force will drive the accumulation and release of the payloads [120]. Previous studies have shown that magnetophoresis can enhance the accumulation and penetration of nanocarriers into solid tumors [122]. However, the clinical translation of this approach is difficult due to its low efficacy and uncontrollable magnetic nanoparticle distribution [123,124]. One of the biggest challenges with the use of magnetic fields is that because the magnetic force falls off significantly with distance, the target sites are limited to the near-surface of the body [120]. Some groups attempted to overcome this challenge by implanting magnets within the body in order to reach deep tissue penetration [120]. However, the use of permanent magnets itself has become a limitation for the clinical translation of this approach [125,126]. In addition, the lack of real-time imaging and the difficulties in controlling magnetic force for precise delivery are also factors that limit its clinical use [125,126]. Using the oppositely polarized magnets, Liu et al. aimed to improve the weak magnetic force in older generations of magnetophoresis that can only be used to treat superficial tumors [122]. This method increased the penetration by five-fold and accumulation by three-fold of magnetic nanoparticles within solid tumors compared to passive enhanced permeability and retention effect [122].

### 4.7. Enzyme-Responsive Nanocarriers

In addition to the environmental stimuli such as pH and oxidative stress, enzymes that present within the cellular system can also be utilized as a trigger for targeted drug release [127]. Nanocarriers such as polymeric micelles, liposomes, and dendrimers are often functionalized by attaching cleavable peptides to the surface that are tailored to specific enzymes present in the targeting tissues [128,129,130,131]. In order to achieve the goal of targeted cargo delivery, several releasing mechanisms can be employed. In functionalized enzyme-responsive liposomes, the enzymes can either directly perturb the lipid bilayer structure, or cleave a lipopeptide or lipopolymer incorporated in the bilayer to achieve destabilization of the nanocarrier [129]. In addition, targeted enzymes can also remove the shielding polymers from the surface to increase cellular uptake, or activate a prodrug in the nanocarriers [129].

Based on the purpose of the treatment, a variety of enzymes can be utilized to trigger local drug delivery. For example, one unique feature of the cancerous tissues is the overexpression of a type of extracellular proteolytic enzyme, called the matrix metalloproteinases (MMPs) [132]. Based on this feature, several MMP-responsive nanocarriers have been developed as one approach for the targeted cancer treatment [133]. For targeted anti-inflammatory treatment, a protease secreted by neutrophils, called human neutrophil elastase (HNE) is exploited as a biological cue for controlled drug release from nanocarriers equipped with HNE-sensitive peptide linkers [134,135]. This approach can significantly increase the sensitivity of targeted anti-inflammatory treatment, as neutrophils are the first cells recruited to inflammatory sites [136]. Enzyme-responsive nanocarriers can also be applied to regulate coagulation locally. For this purpose, Bhat et al. developed a thrombin-responsive mesoporous silica nanoparticle (MSN) that is loaded with an anticoagulant drug and capped with a peptide containing a thrombin-specific cleavage site [137]. When the coagulation cascade is triggered, active thrombin can degrade the capping peptide sequence on the nanocarrier and release the anticoagulant locally [137]. Due to the site-specificity of thrombin, thrombin-responsive nanocarriers possess the advantage of spatiotemporal specificity in anti-thrombotic drug delivery [138].

While significant progress has been made in the development of enzyme-responsive nanocarriers, several challenges remain to be addressed for the wider application of this approach. For example, there are various enzyme subtypes existing in the biological system that share similar cleavage sites [130]. In addition, current imaging technologies are yet to satisfy the need for the confirmation of controlled drug release at the targeted areas [130].

### 4.8. Multimodal Nanocarriers

Despite the fact that stimuli-responsive nanocarriers have already significantly improved targeted drug delivery, they still face certain limitations. For example, stimuli-responsive nanocarriers utilizing external factors, such as ultrasound, light, heat, and magnetic forces, are limited to targets with known target localization. In addition, human physiology is a complex system. Nanocarriers that are responsive to pH and oxidative stress might potentially release cargoes at areas other than the diseased area because those non-targeted areas also share similarly elevated pH or oxidative stress, due to conditions such as metabolic acidosis, vascular occlusion, or other inflammations. By combining different stimuli-responsive properties, multimodal nanocarriers can improve the efficacy of nanomedicine, especially in cancer therapy [139]. Recently, nanocarriers that possess both thermo- and pH-responsiveness are being developed to treat certain cancers [140]. Using this dual stimuli-responsive system, Hiruta et al. developed a polymeric micelle for anti-cancer drug delivery that can be selectively up-taken with external thermal stimulation and effectively release its cargo at endosomal pH [141]. As each stimuli-responsive nanocarrier has different advantages and limitations (Table 2), with additional stimuli responsiveness, nanocarriers can be more attuned to the stimuli changes and exert therapeutic effects in a more precise fashion. Recently, a programmable polymer library for stimuli-responsive nanocarriers containing logic gates has also been developed to gather systemic information in order to achieve precision medicine [142,143].

Another example in the anticancer drug delivery system is the nanocarriers designed to respond to the tumor microenvironment which can switch its size and morphology in response to the acidic tumor microenvironment and near-infrared laser irradiation [144]. Jia et al. have recently developed a smart nanodrug that can switch its size and morphology in response to the acidic tumor microenvironment and near-infrared laser irradiation to effectively ablate a tumor, inhibiting tumor metastasis [144]. This nanodrug is assembled by a cytolytic peptide, an NIR-absorbing molecule, and a tumor-targeting polymer [144]. Under normal physiological environment, the assembly is a negatively charged nanosphere about 50 nm in size [144]. The acidic tumor microenvironment triggers the transformation of the nanodrug into net-like nanofibers [144]. The net-like structure helps to limit the mobility of tumor cells and also prolongs the drug retention time [144]. During photothermal therapy, the nanocomplex can be photodegraded into smaller nanospheres about 25 nm in size to allow deeper tumor penetration of the drug [144].

When combining gene and photothermal therapy, synergistic therapeutic effects have been observed, suggesting the advantages of multimodal nanomedicine [145]. In a similar effort for on-demand drug release, Deng et al. developed a new liposomal drug delivery platform that can control payload release only when triggered by x-ray radiation [146]. This liposome incorporates gold nanoparticles with a photosensitizer called verteporfin [146]. Under radiation, the photosensitizer produces singlet oxygen to destabilize the liposomal membrane, allowing payload to be released from the liposome, while the gold nanoparticles are used for radiation enhancement [146]. This platform could provide synergistic therapeutic effects in chemotherapy when combined with radiotherapy [146]. The main drawback of this platform design, however, is that the photosensitizer, as well as the gold nanoparticle incorporated, generate a certain level of ROS in the tissues that could be damaging [146].

Continued efforts are being made to advance theranostic nanocarriers. By functionalizing the surface of FDA-approved iron oxide nanoparticles with an imaging contrast agent and a peptide activatable by a tumor-specific enzyme new theranostic nanocarriers can achieve enzyme-specific drug delivery at the site of the tumor and simultaneous MRI imaging [147]. Conjugated polymer nanosystems are developed to combine diagnostic imaging together with photothermal therapy and drug delivery in cancer therapy [148]. Li et al. have also functionalized the carbon quantum dots so that they structurally mimic large amino acids that can selectively accumulate at tumor sites for both imaging and drug delivery purposes [149]. A recent review by Riccardi et al. summarizes the development of different nanocarriers with various decorations (or functionalization) for the improvement in bioavailability, pharmacokinetics, and specificity of anticancer ruthenium-based drugs [150].

### 4.9. Bioinspired Nanocarriers

Besides using biodegradable and biocompatible synthetic materials for the development of nanocarriers, other bioinspired natural materials are also being explored for their application in drug delivery systems. One strategy is to develop biomimetic nanoparticles by using the cell membrane as camouflage [151]. For example, utilizing the concept of biomimetic functionalization of the nanocarriers, Liu et al. integrated a red blood cell (RBC) membrane vesicle with near-infrared persistent luminescence nanophosphors to ensure the nanocarriers can bypass macrophage uptake and systemic clearance to improve circulation time for bio-imaging and drug delivery [152]. Santos’ group further engineered the isolated RBC membranes to form nanoerythrosomes (NERs), i.e., derivatives of RBCs with an average diameter of 100 nm, for drug delivery [153]. Similarly, using a cancer membrane as camouflage, a tumor homing nanocarrier that carries imaging and/or therapeutic moieties can also provide a new platform for targeted drug delivery [154,155]. Compared to RBC membranes, cancer cell membranes alone are unstable and have insufficient drug entrapment, thus cannot act as an autonomous drug delivery system without the support of other nanomaterials [153]. Therefore, Balasubramanian et al. combined cancer cell membrane material with porous silicon nanoparticles to develop nanocarriers that serve as artificial organelles in order to supplement cellular functions under oxidative stress [156].

In addition to using bioinspired nanomaterials to develop new nanocarriers, existing nanocarriers can also be modified in a bioinspired fashion. Zhang et al. recently developed a liposomal nanocarrier with a modified surface that has a short nontoxic peptide derived from Aβ1-42 that specifically interacts with the lipid-binding domain of apolipoproteins [157]. These nanocarriers absorb plasma apolipoproteins A1, E, and J, resulting in the exposure of the receptor-binding domain of apolipoproteins to achieve brain-targeting drug delivery [157].

## 5. Strategies to Enhance Therapeutic Efficacy Using Different Payloads

### 5.1. Cell Replacement

Nanocarriers can also deliver other payloads such as cells to the diseased area. Such an approach can be applied in cell replacement therapies. For example, for type I diabetic patients, islet transplantation is a promising treatment [158]. However, it is limited by the shortage of donors and the significant side effect of immunosuppression [158]. In order to overcome this challenge, recent advancements in nanotechnology enables the encapsulation of the islet in immune-isolating membranes with chemical modifications for transplantation [29,158,159]. Using mesenchymal stem cells (MSCs) as cell-based drug delivery vectors for tumor-homing cancer treatment has also shown some promising results [160]. However, the broad biodistribution of MSCs also raises concerns for toxicity to non-target peripheral tissues [160]. A wider application of nanocarrier assisted cell replacement therapy still requires more investigation.

### 5.2. Gene Therapy

Another area of investigation that is currently on the horizon is the concept of nanocarrier assisted gene therapy. One important strategy in gene therapy is the use of small interfering RNA (siRNA) to silence disease-causing genes [161]. Currently, there are more than 20 siRNA based therapies in clinical trials [161]. However, there are two main concerns of RNA interference (RNAi) potency and specificity [162]. Transporting siRNA across the cell membrane is challenging due to its anionic property [163]. In addition, naked siRNA has immunostimulatory effects and is easily degraded in the bloodstream [163]. Viral vectors have long been used to deliver siRNA, however, they have drawbacks, such as being immunogenic and cytotoxic [164]. Being non-viral and equipped with targeting capacity, nanocarriers have drastically helped the emergence of RNAi therapeutics [161]. Different nanocarriers are being applied in the delivery of siRNA, such as nucleotides, lipids, and polymers [161,163]. The biocompatibility and design flexibility of nanocarriers allow better control of siRNA delivery to achieve desired gene knockdown efficiency [162,163,164].

Among all the nanocarriers under development for their potential to assist in gene delivery, lipid nanocarriers have shown the most promising results in the clinical translation of siRNA therapy [165,166,167,168,169]. Lipid nanoparticles can protect siRNA from degradation, and facilitate endocytosis and endosomal escape [168]. The first nanoparticle assisted targeted RNAi delivery in humans was reported in 2009 [170]. In this study, the nanocarriers were designed to passively accumulate and permeate in solid tumors [170]. With the help of CRISPR-Cas9 technology, new lipid nanocarriers capable of selective organ targeting (SORT) have been developed [171]. These nanocarriers can target extrahepatic tissues with the aid of targeting molecules for selective organs, revolutionizing tissue-specific gene editing [171]. Lipid nanocarrier assisted nucleic acid delivery is also under active investigation for its potential use in the development of prophylactic vaccines [172]. In addition, recently another type of nanocarrier similar to the liposome, called noisome, has also been developed [173]. Niosomes are self-assembled vesicles made up of single-chain non-ionic surfactants combined with appropriate amounts of cholesterol or other lipids [173]. Similar to liposomes, niosomes are capable of carrying hydrophilic or lipophilic drugs but are more stable, less expensive, and easier to manipulate [173]. They have the potential to be an alternative gene delivery system.

## 6. Safety

With a growing number of nanomedicines receiving FDA approvals for their clinical use, a proper evaluation of the safety profile of the nanomedicine becomes increasingly important. However, the majority of assays currently available for safety assessment were developed to test conventional therapeutics [174]. For example, the toxicity assessment of oral nanocarriers still depends on in vitro experiments using different cell lines [175]. In response to the unique size and shape of nanoparticles, more suitable assays need to be employed for a proper safety evaluation [174]. For example, unlike conventional drugs, some nanocarriers are not able to diffuse through the cell membrane due to their polarity but enter cells via endocytosis [176,177]. A safe entry of the cells, therefore, becomes a crucial factor to be considered for the effectiveness of nanomedicine [176]. Since macrophages are considered to be the first cells that take up nanoparticles, assessing the impact of nanocarriers on macrophages can be used to evaluate their immunocompatibility [178]. Mottas et al. have recently developed a rapid screening method to evaluate the impact of nanoparticles on macrophages, providing a new approach to assess cytotoxicity as well as quality control of nanoparticles [178]. Rapid screenings could also ease the process for the scaled-up production of nanocarriers [178].

The interaction of nanoparticles with blood components also provides important information regarding the safety profile of the nanoparticles. Fornaguera et al. conducted a variety of testing methods to assess the safety profile of a biocompatible nanomaterial, PLGA, when interacting with blood components [179]. They found that fibrinogen aggregation was dependent on the surface charge of nanoparticles [179]. Complement activation was influenced by the functionalization and concentration of nanoparticles [179]. Based on the results from their study, PLGA is considered safe concerning embolism or cell lysis [179].

However, Howard et al. raised a very important issue that should be considered when developing new nanocarriers, that is, the biocompatibility of a single component does not guarantee the safety of the nanocarrier [180]. Even for nanocarriers composed of biocompatible materials and carrying benign cargos, it is still possible that they may elicit pro-inflammatory effects [180]. Many unintended side effects may occur in major organs due to the size, shape, or charge of the nanocarriers [181]. Therefore, systemic effects of the nanocarriers, such as activation of complement, coagulation or the platelets, and toxicity toward the clearing tissues (liver, kidney, lungs, etc.), must be assessed in vivo before further application [180]. In order to alleviate the side effects when interacting with host defenses, Parhiz et al. also suggested several approaches as alternative modification or functionalization of those nanocarriers. For example, using hydroxyethyl starch (HES), polysialic acid, dextrin, and poly(phosphoester)s (PPEs) as an alternative for PEG, because PEG-specific antibodies have been found to be generated following administration of PEGylated liposomes, accelerating the clearing process of those liposomes [181]. Other suggestions include replacing the antibodies with safer fragments or inducing immune tolerance of the host using various approaches [181].

With the rising awareness of assessing the safety profiles of newly developed nanocarriers, more and more investigators have started including in vivo safety and toxicity assessments in their studies. Common evaluations include: (1) hemolysis, platelet activation, and inflammatory responses using completed blood counts, white blood cell differential counts, and specific assays, (2) functional tests such as renal function studies and liver function tests, as well as (3) the potential tissue damage assessment in major organs using histology [133,182].

Additionally, new efforts are also being made to assess the subchronic and chronic toxicity of inorganic nanoparticles, including iron oxide [183]. Chronic safety evaluation of the nanocarriers is important because a common concern over the use of nanocarriers is that the oxidation of the corona protein formed during the interaction between the nanoparticle and the physiological environment will induce oxidative stress to the cells, and the chronic oxidative stress imbalance will exert harmful effects [184,185].

As careful evaluations of the safety profile of each nanocarrier are necessary prior to their clinical applications, interdisciplinary collaborations among researchers in nanotechnology and biomedicine are highly encouraged in order to ensure a safe development of nanomedicine.

## 7. Clinical Landscape and Challenges

Following the first FDA approval of PEGylated liposomal Doxorubicin, more nanocarrier assisted pharmaceutics received FDA approval for their clinical use in recent years [10]. Study entries on Clinicaltrial.gov showed that so far there are 140 completed clinical trials using nanoparticles, of which, only 37 trials on drug delivery shared their results. While the approved nanomedicines cover several areas of medicine, cancer therapy still attracts much of the research interest. For example, VYXEOS is a liposomal nanocarrier encapsulating two anti-cancer drugs, daunorubicin and cytarabine, for the treatment of acute myeloid leukemia [186]. Compared to previously approved liposomal nanocarriers, which only carry one drug per nanocarrier, VYXEOS advanced the field by carrying two synergistic chemotherapeutics to improve the therapeutic efficiency [186]. In the clinical trials leading up to the approval of VYXEOS, patients receiving VYXEOS had a higher survival rate compared to those who received nanocarrier-free drugs, because higher therapeutic efficacy and lower toxicity were achieved in the VYXEOS group [186].

Gene therapy has also advanced significantly towards clinical translation in recent years. This is reflected in the recent approval of patisiran (ONPATTRO™), which is another lipid nanocarrier that carries a double-stranded siRNA that specifically inhibits hepatic synthesis of transthyretin, the disease-causing protein of hereditary transthyretin amyloidosis [187]. Results from the APOLLO trial (NCT01960348) showed that patisiran was able to improve multiple clinical manifestations of hereditary transthyretin amyloidosis [188]. The examples of FDA approved nanomedicine revealed that the majority of the approved nanocarriers are liposomal carriers via the intravenous infusion route [186]. In order to improve the patients’ quality of life, there are also lipid-based nanocarriers that are modified to resist the gastro-intestinal environment under development for oral peptide delivery [189]. Anselmo et al. nicely summarized the recently approved nanocarriers since 2016 [186]. In addition, records on clinicaltrials.gov have also shown that there are many nanocarriers currently in phase II or III clinical trials (Table 3). Especially, clinical trials using liposomal doxorubicin are still currently dominating the market.

Several challenges still hinder the application of nanomedicine. One obstacle is the protein corona. When nanoparticles enter human bodies, the interaction between nanoparticles and the physiological environment creates protein corona around the nanoparticle [157]. Serum albumin was found to be adsorbed onto all types of nanoparticles, even for nanocarriers made of biodegradable materials such as PLGA [179]. The protein corona is a major obstacle for the bench-to-bedside translation of targeted drug delivery systems using nanocarriers as it induces unfavorable biodistribution [157]. Recent technological advances have improved the stability of nanocarriers using various surface modifications [190]. However, much more improvement still needs to be done for a safer application of nanocarriers.

New discoveries in nanomedicine also help us challenge the old paradigm to make breakthroughs. The paradigm that has long been established regarding how nanoparticles enter solid tumors is that they enter through the gaps of the tumor vasculatures [191]. After continuous efforts to translate the preclinical results into clinical use, the notion of passive permeabilization and accumulation in tumors came into question [191]. Most recently, Chan’s group reexamined the entry of nanoparticles into solid tumors [191]. Using four different models, they found that tumor vasculature is mostly continuous and has a very low gap frequency, and up to 97% of nanoparticles enter solid tumors via an active process through endothelial cells, and passive extravasation contributed only a small fraction of the nanoparticle tumor accumulation [191]. Such a finding challenges the current paradigm about nanoparticle entry and encourages the refined design of the nanoparticle to improve targeting efficiency and drug delivery efficacy rather than merely depending on the passive accumulation. Moving forward, some areas need further investigation in order to overcome currently poor translational outcomes of nanomedicine, such as identifying the mechanisms of nanoparticles’ interaction with different tissues and their uptake in the body [191].

From the production point of view, despite the advances in FDA approvals, large scale production of nanomedicine is still very challenging. Several factors hindering the progression include low loading efficiency, difficulty in homogeneous production, and purification [16]. Moreover, the size of the nanoparticles is an important characteristic because it can impact the absorption, biodistribution, and excretion of the nanoparticles [16]. However, the nanoscale size range is a particularly difficult region for appropriate method selection, being restrained by either the upper or lower resolution limit of many instruments [16]. In the meantime, the types and complexity of these nanoparticles have increased over the years and are expected to further increase in the coming years [16].

## 8. Conclusions

In this review, we discussed different types of nanocarriers applied in the drug delivery system. The use of nanocarriers, whether with single-featured nanomaterials and/or structures or in a hybrid fashion, has largely expanded the platform for drug delivery. In addition, the functionalization of nanocarriers that enables them to be sensitive to different stimuli (such as pH, heat, light, or oxidative stress) has further broadened their capacities for the delivery of different therapeutics. While tremendous advancements have been achieved in this field over the years, challenges remain to overcome in order to improve the translational value of current nanomedicine research. Specifically, the long-term safety profiles of these nanocarriers need to be carefully evaluated. Looking ahead, the nanocarrier assisted-delivery system holds great potential in improving therapeutic efficacy.

## Figures and Tables

**Table 1:** Features of different types of nanocarriers.

Nanocarrier Type	Advantages	Challenges/Limitations	Safety Concerns	References
Liposome	Biocompatible Easily modifiable Large drug loading capacity Self-assembly	Fast clearance Off-target accumulation	Good safety profile	[10,11,12,16,17]
Biodegradable Polymeric Micelle	Versatile compositions Biocompatible Self-assembly Prolonged therapeutic efficacy	Relatively low drug loading capacity May reduce the effectiveness of the conjugated drug	Good safety profile	[23,24,25,26,27]
Carbon Nanotube (CNT)	Easily internalized by cells	Insoluble unless functionalized	Morphology induced toxicity Surface charge-dependent toxicity	[40,41,42,43,44,45,46]
Carbon Dot (CD)	High drug loading yield Better controllability in drug conjugation High fluorescent quality Easily internalized by cells	Slow drug release May reduce the effectiveness of a conjugated drug	Toxicity depends on surface charge PEGylated CDs that are neutrally charged present to be safe	[49,50,51,52,55,56]
Iron Oxide Nanoparticle	Potent magnetic and catalytic properties Biocompatibility High drug loading capacity	Performance directly related to size, shape, and surface charge	Conflicting toxicity evidence ROS generation	[57,58,60,61,62]
Nanogel	High biocompatibility High drug loading capacity High water content Tunable size and stability	Fast clearance Off-target accumulation Surface charge Difficulties in controlling both degradation and drug release Drug loading and targeting capacity may change when deformity occurs	Good safety profile	[63,64,65,66,67]
Dendrimer	High surface to volume ratio Capacity to entrap large molecular weight hydrophilic/hydrophobic entities	Fast clearance Off-target accumulation	Toxicity issues with cationic dendrimers	[68,69,70,72]

**Table 2:** Summary of different stimuli-responsive nanocarriers.

Stimuli Type	Advantages	Challenges/Limitations	References
Thermo-Responsive	Tunable drug release	The clearance and accumulation of NPs in off-target organs such as the liver and spleen Limited to the targets with known localization	[80,83,84]
pH-Sensitive	Localized delivery Reduced side-effects Prolonged activity	Fast clearance Specificity limited to the microenvironment	[80,86,87,88,90,91,92,93,94,95]
Ultrasound-Triggered	On-demand drug release Enhanced drug accumulation Non-invasive	Production of intracellular free radicals in response to ultrasound Limited to the targets with known localization	[80,96,97,98]
Light-Responsive	Little to no leakage in the absence of a trigger High efficiency in drug releasing	Non-sustained drug release, might require multiple administration Side-effects of byproducts DNA damage by UV light Limited to the targets with known localization	[99,100,101]
Redox-Responsive	Localized delivery Prompt response in cargo release	Quickly oxidized Fast clearance Specificity limited to the microenvironment	[103,105,106]
Magnetic-Targeting	Improved targeting efficiency	Energy consuming Selection of the proper magnetic carrier and magnetic force Low therapeutic efficacy Difficult to control magnetic nanoparticle distribution and deep tissue penetration Limited to the targets with known localization	[122,123,124,125,126]
Enzyme-Responsive	Localized delivery Higher therapeutic efficacy	Less controllable drug release Difficulty in constructing enzyme-specific substrate	[127,128,129,130,131,133]

**Table 3:** Examples of nanocarriers currently on phase II/III clinical trials.

Study Title	Type of Nanocarrier	Condition	Mechanisms	Status
Repeated DermaVir Immunizations in HIV-1 Infected Treatment-naïve Patients (GIEU006)	Pathogen-like plasmid DNA polymeric nanoparticle [192]	HIV infection	Langerhans cells with DermaVir migrate to lymph nodes and induce HIV-specific T cells that can kill HIV-infected cells	Phase II completed
Neurotoxicity Characterization Study of Nab-paclitaxel Versus Conventional Paclitaxel in Metastatic Breast Cancer (neurabrax)	Nanoparticle albumin-bound drug [193]	Breast Cancer	Exploit natural albumin pathways to enhance the selective uptake and accumulation of anti-cancer drug at the site of the tumor, thus reducing its diffusion to normal tissues.	Phase II completed
Combination Therapy With NC-6004 and Pembrolizumab in Head and Neck Cancer Subjects Who Have Failed Platinum Regimen	Micellar nanoparticles [194]	Squamous Cell Carcinoma of the Head and Neck	The hydrophilic nature of micelle increases the water-solubility of the anti-cancer drug and decreases the nephrotoxicity and neurotoxicity associated with the administration of drug alone.	Phase II recruiting
Clinical Assessment of Voriconazole Self Nano Emulsifying Drug Delivery System Intermediate Gel	Nanolipid in situ gel [195]	Tinea Versicolor infection	Solid lipid nanoparticles are incorporated into in situ gels for sustained release of the drug, to prolong the residence time, and to increase the bioavailability of the drug.	Phase II completed
